# Prognostic Value of Leukocytosis and Lymphopenia for Coronavirus Disease Severity

**DOI:** 10.3201/eid2608.201160

**Published:** 2020-08

**Authors:** Glen Huang, Alex J. Kovalic, Christopher J. Graber

**Affiliations:** David Geffen School of Medicine at University of California Los Angeles, Los Angeles, California, USA (G. Huang, C.J. Graber);; Novant Forsyth Medical Center, Winston Salem, North Carolina, USA (A.J. Kovalic);; VA Greater Los Angeles Healthcare System, Los Angeles (C.J. Graber)

**Keywords:** 2019 novel coronavirus disease, coronavirus disease, COVID-19, severe acute respiratory syndrome coronavirus 2, SARS-CoV-2, viruses, respiratory infections, zoonoses, leukocytosis, lymphopenia, meta-analysis

## Abstract

To evaluate lymphopenia as a marker for coronavirus disease severity, we conducted a meta-analysis of 10 studies. Severe illness was associated with lower lymphocyte and higher leukocyte counts. Using these markers for early identification of patients with severe disease may help healthcare providers prioritize the need to obtain therapy.

The incidence of coronavirus disease (COVID-19), caused by severe acute respiratory syndrome coronavirus-2 (SARS-CoV-2), has spread rapidly globally; as of March 29, 2020, ≈670,000 cases had been confirmed worldwide (*1*). COVID-19 is typically a pulmonary infection that can range from mild illness to acute respiratory distress syndrome and multiple organ failure; however, other symptoms such as myalgias and anorexia have been noted (*2*). Although many ongoing studies are investigating measurement of proinflammatory cytokines and other biomarkers as a way to prognosticate infection severity, we investigated use of 2 easily obtained predictors: lymphopenia and leukocytosis (*3*).

## The Study 

We searched 3 major databases—MEDLINE/PubMed, EMBASE, and CENTRAL (Cochrane Central Register of Controlled Trials)—for clinical studies published December 1, 2019, through March 28, 2020. To broadly identify studies detailing lymphocyte and leukocyte testing among patients with COVID-19, we used the following search criteria: “(COVID-19 OR SARS-CoV-2 OR 2019-NCoV OR HCov-19 OR novel coronavirus) AND (laboratory OR WBC OR lymphocyte).” We prioritized studies that measured lymphocyte and leukocyte counts among patients who had severe or critical cases versus those with mild cases. Severe cases were defined as significant respiratory distress (acute hypoxic respiratory failure, acute respiratory distress syndrome, need for mechanical ventilation, or intensive care unit admission) caused by COVID-19.

Our meta-analysis included articles about studies and clinical trials that met the following 4 inclusion criteria: 1) involved adult, human patients; 2) written in English; 3) reported lymphocyte and leukocyte counts for patients; and 4) compared patients with severe versus mild illness. Our meta-analysis excluded articles about studies with the following 9 characteristics: 1) involved nonhuman subjects; 2) written in a language other than English; 3) were not a clinical trial, such as a review paper or letter; 4) were out of the scope of the study question detailed above; 5) did not provide raw data to perform quantitative meta-analysis; 6) involved pediatric patients; 7) did not have the full article available; 8) were duplicates; and 9) were ongoing or not completed.

We performed the meta-analysis by using Review Manager software version 5.3 (The Cochrane Collaboration, https://training.cochrane.org). We calculated mean differences (MDs) between groups for continuous variables and reported 95% CIs for both severe and nonsevere cases. If the included studies provided medians and interquartile ranges instead of MDs and SDs, we imputed the MDs and SDs as described previously (*4*–*6*) and additionally described in the Cochrane Handbook for Systematic Reviews (*7*). We considered results statistically insignificant if p>0.05 or if the MD included zero. We assessed statistical heterogeneity by using the *I^2^* statistic and awarded the following values: 0%–24%, homogeneity; 25%–49%, mild heterogeneity; 50%–74%, moderate heterogeneity; and >75%, high heterogeneity. If moderate to high heterogeneity was present (*I*^2^>50%), then we used the random effects model to pool the effect sizes of included studies and subgroup analyses.

We identified 959 articles; among them, 318 were duplicates. We then screened the remaining 641 by title, abstract, or both. We assessed 59 articles as eligible (included patient-level clinical data) and identified 8 studies (included lymphocyte counts and stratification of illness severity) for the quantitative synthesis (Appendix Table). These studies described 1,289 cases of COVID-19, of which 592 (45.9%) were classified as severe. 

We compared lymphocyte and leukocyte counts in patients with severe/critical versus mild cases of COIVD-19 (Appendix Figure). All laboratory data were captured at the time of patient admission. Overall, patients categorized as having severe illness tended to have lower lymphocyte counts (pooled MD −0.36, 95% CI −0.50 to −0.22; p<0.00001) and higher leukocyte counts (pooled MD 1.32, 95% CI 0.62 to 2.02; p<0.00001). Fan et al. reported an absolute lymphocyte count of >1.0 × 10^9^ cells/L for 39/58 (69.6%) patients in the nonsevere group and 2/9 (22.2%) patients in the severe group (*8*). Huang et al. reported an absolute lymphocyte count of <1 × 10^9^ cells/L in 15/28 (54%) patients in the nonsevere group and 11/13 (83%) in the severe group (*9*). Wan et al. reported an absolute lymphocyte count of <1.0 × 10^9^ cells/L for 36/95 (38%) patients in the nonsevere group and 32/40 (80%) in the severe group (*10*). Zhang et al. reported a decreased lymphocyte count for 28/82 (70.7%) patients in the nonsevere group and 46/56 (82.1%) in the severe group (*11*).

## Conclusions 

Pooled data across early studies validate a significant correlation between elevated leukocyte count and decreased lymphocyte count among patients with severe cases of COVID-19 compared with those with mild cases. Why lymphopenia is associated with severe illness remains unclear. It has been hypothesized that this association could result from direct lymphocyte infection, destruction of lymphatic tissue, inflammation leading to lymphocyte apoptosis, or inhibition of lymphocytes by metabolic disorders such as lactic acidosis (*12*). Lymphopenia as a marker of severity does not seem to be specific to COVID-19; it has been used to prognosticate other viral pneumonias such as influenza (*13*). Neutrophilia may be more specific to severe disease than leukocytosis, but neutrophil count was not uniformly reported across the studies included in our analysis.

Despite our findings regarding clinical characteristics of severe COVID-19, our study had several limitations. First, our literature search found an expected paucity of data surrounding this topic because published characterizations of patients with COVID-19 remain minimal. More COVID-19 data from other nations and patient populations will aid in the comparison and validation of our clinical findings. Second, we noted significant heterogeneity in both the leukocyte and lymphocyte analyses. This phenomenon probably resulted from the small sample size, limited and early data, and skewed representation of the patient population. Third, the definitions of severe cases were somewhat inconsistent across these studies, varying from acute hypoxic respiratory failure to requiring mechanical ventilation. This variability could further compound the heterogeneity found across these studies. Last, only a minority of the manuscripts reported the proportion of patients with lymphopenia, and variable cutoffs based on the articles’ reference ranges made it difficult to ascertain a cutoff for severe disease. 

With the rising cases of COVID-19 and limited resources (*14*), being able to prioritize patients with severe disease is crucial. Some therapeutic agents are being investigated (*15*); however, supplies are often low and procurement may be delayed. The sooner patients with severe disease can be identified, the sooner the process of obtaining therapy can be initiated.

Appendix Additional information for prognostic value of lymphocyte and leukocyte values in patients with severe and nonsevere coronavirus disease.
