# Supplementary material for: Prognostic Value of Leukocytosis and Lymphopenia for Coronavirus Disease Severity
Source: Emerg Infect Dis. 2020 Aug;26(8):1839–41. doi: 10.3201/eid2608.201160 (PMC7392413; doi:10.3201/eid2608.201160)
Supplement: Appendix — Additional information for prognostic value of lymphocyte and leukocyte values in patients with severe and nonsevere coronavirus disease. [file 20-1160-Techapp-s1.pdf]

Article DOI: https://doi.org/10.3201/eid2608.201160

Prognostic Value of Leukocytosis and Lymphopenia for Coronavirus Disease Severity

Appendix

Appendix Table. Studies included in quantitative meta-analysis of leukocytosis and lymphopenia in non-severe vs. severe COVID-19 disease

	Fan et al (*1*)		Huang et al (*2*)		Qian et al (*3*)		Qin et al (*4*)		Wan et al (*5*)		Wang et al (*6*)		Wu et al (*7*)		Zhang et al (*8*)	
Characteristics	Non-severe	Severe *****	Non-severe	Severe *****	Non-severe	Severe	Non-severe	Severe	Non-severe	Severe	Non-severe	Severe *****	Non-severe	Severe †	Non-severe	Severe
Location	Singapore		Wuhan, China		Ningbo, China		Wuhan, China		Chongqing, China		Wuhan, China		Wuhan, China		Wuhan, China	
Cases	58	9	28	13	82	9	166	286	95	40	102	36	117	84	82	58
Demographics																
Age in years (IQR)	41 (32,53)	54 (47,62)	49 (41.57.5)	49 (41,61)	49 (35.3,56)	66 (54–80)	43 (41.25,62)	61 (51,69)	44 (33,49)	59 (52,73)	51 (37,62)	66 (57,78)	48 (40,54)	58.5 (50,69)	51.5 (26,78)	64 (25,87)
Women (%)	27 (48.6%)	3 (33.3%)	9 (32%)	2 (15%)	NR	NR	86 (51.8%)	131 (45.8%)	43 (45.3%)	19 (47.5%)	49 (48%)	14 (38.9%)	49 (41.9%)	24 (28.6%)	44 (53.7%)	25 (43.1%)
Laboratory data																
Leukocyte x 109/L (IQR)	4.7 (4,5.8)	5.1 (3.5,8.2)	5.7 (3.1,7.6)	11.3 (5.8, 12.1)	4.97 (4.02,5.65)	5.23 (4.74,6.8)	4.9 (3.7,6.1)	5.6 (4.3,8.4)	5.5 (4,8)	5.2 (4.9,6.9)	4.3 (3.3,5.4)	6.6 (3.6–9.8)	5.02 (3.37,7.18)	8.32 (5.07,11.20)	4.5 (3.5,5.9)	5.3 (4,9)
Lymphocyte x 109/L (IQR)	1.3 (0.9,1.7)	0.5 (0.48,0.8)	1 (0.7,1.1)	0.4 (0.2,0.8)	1.4 (1.05,1.75)	0.9 (0.7,1.3)	1 (0.7,1.3)	0.8 (0.6,1.1)	1.2 (0.8,1.6)	0.8 (0.6,1)	0.9 (0.6–1.2)	0.8 (0.5–0.9)	1.08 (0.72,1.45)	0.67 (0.49,0.99)	0.8 (0.6,1.2)	0.7 (0.5,1)
Lymphocyte % (IQR)	NR	NR	NR	NR	NR	NR	21.4 (15.3–32.5)	14.1 (8.8–21.4)	NR	NR	NR	NR	NR	NR	20 (12.5–28.4)	12.7 (7.7–22)
Neutrophils x 109/L (IQR)	2.6 (2.1,3.8)	4.2 (2.1,6.9)	4.4 (2,6.1)	10.6 (5,11.8)	2.8 (2.18,3.49)	3.32 (3,5.82)	3.2 (2.1–4.4)	4.3 (2.9,7)	3.6 (3,3.9)	4.1 (3.1,5.7)	2.7 (1.9,3.9)	4.6 (2.6,7.9)	3.06 (2.03,5.56)	1.04 (3.98,10.12)	NR	NR

*Severe defined as admitted to the intensive care unit.
†Severe defined as having acute respiratory distress syndrome.

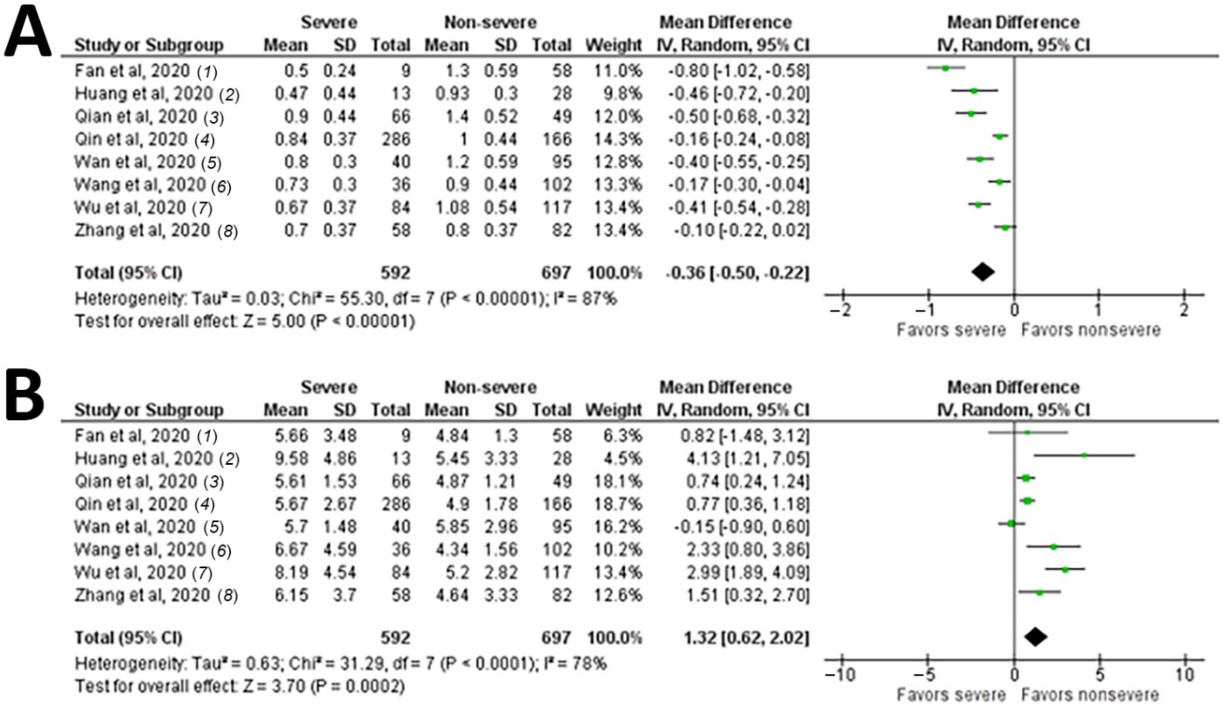

Figure. Forest plot of lymphocyte (A) and leukocyte (B) values in patients with severe and nonsevere coronavirus disease.

References

1. Fan BE, Chong VCL, Chan SSW, Lim GH, Lim KGE, Tan GB, et al. Hematologic parameters in patients with COVID-19 infection. Am J Hematol. 2020;ajh.25847. https://doi.org/10.1002/ajh.25847

2. Huang C, Wang Y, Li X, Ren L, Zhao J, Hu Y, et al. Clinical features of patients infected with 2019 novel coronavirus in Wuhan, China. Lancet. 2020;395:497–506. PubMed https://doi.org/10.1016/S0140-6736(20)30183-5

3. Qian GQ, Yang NB, Ding F, Ma AHY, Wang ZY, Shen YF, et al. Epidemiologic and Clinical Characteristics of 91 Hospitalized Patients with COVID-19 in Zhejiang, China: A retrospective, multi-centre case series. QJM. 2020;hcaa089. PubMed https://doi.org/10.1093/qjmed/hcaa089

4. Qin C, Zhou L, Hu Z, Zhang S, Yang S, Tao Y, et al. Dysregulation of immune response in patients with COVID-19 in Wuhan, China. Clin Infect Dis. 2020 Mar 12. https://doi.org/10.1093/cid/ciaa248

5. Wan S, Xiang Y, Fang W, Zheng Y, Li B, Hu Y, et al. Clinical features and treatment of COVID-19 patients in northeast Chongqing. J Med Virol. 2020. PubMed https://doi.org/10.1002/jmv.25783

6. Wang D, Hu B, Hu C, Zhu F, Liu X, Zhang J, et al. Clinical characteristics of 138 hospitalized patients with 2019 novel coronavirus-infected pneumonia in Wuhan, China. JAMA. 2020;323:1061–9. https://doi.org/10.1001/jama.2020.1585

7. Wu C, Chen X, Cai Y, Xia J, Zhou X, Xu S, et al. Risk factors associated with acute respiratory distress syndrome and death in patients with coronavirus disease 2019 pneumonia in Wuhan, China. JAMA Intern Med. 2020 Mar 13:e200994. https://doi.org/10.1001/jamainternmed.2020.0994

8. Zhang JJ, Dong X, Cao YY, Yuan YD, Yang YB, Yan YQ, et al. Clinical characteristics of 140 patients infected with SARS-CoV-2 in Wuhan, China. Allergy. 2020 Feb 19 [Epub ahead of print]. https://doi.org/10.1111/all.14238
